# Marital Status, Living Arrangement, and Cancer Recurrence and Survival in Patients with Stage III Colon Cancer: Findings from CALGB 89803 (Alliance)

**DOI:** 10.1093/oncolo/oyab070

**Published:** 2022-02-19

**Authors:** Seohyuk Lee, Chao Ma, Sui Zhang, Fang-Shu Ou, Tiffany M Bainter, Donna Niedzwiecki, Leonard B Saltz, Robert J Mayer, Renaud Whittom, Alexander Hantel, Al Benson, Daniel Atienza, Hedy Kindler, Cary P Gross, Melinda L Irwin, Jeffrey A Meyerhardt, Charles S Fuchs

**Affiliations:** Yale School of Medicine, New Haven, CT, USA; Department of Medical Oncology, Dana-Farber Cancer Institute, Boston, MA, USA; Department of Medical Oncology, Dana-Farber Cancer Institute, Boston, MA, USA; Alliance Statistics and Data Management Center, Mayo Clinic, Rochester, MN, USA; Alliance Statistics and Data Management Center, Mayo Clinic, Rochester, MN, USA; Department of Biostatistics and Bioinformatics, Duke University, Durham, NC, USA; Memorial Sloan Kettering Cancer Center, New York, NY, USA; Department of Medical Oncology, Dana-Farber/Partners CancerCare, Boston, MA, USA; Hôpital du Sacré-Coeur de Montréal, Montreal, Canada; Loyola University Stritch School of Medicine, Naperville, IL, USA; Robert H. Lurie Comprehensive Cancer Center, Northwestern University, Chicago, IL, USA; Virginia Oncology Associates, Norfolk, VA, USA; University of Chicago Comprehensive Cancer Center, Chicago, IL, USA; Yale School of Medicine, Department of Internal Medicine, New Haven, CT, USA; Yale School of Public Health, New Haven, CT, USA; Department of Medical Oncology, Dana-Farber/Partners CancerCare, Boston, MA, USA; Yale Cancer Center, Smilow Cancer Hospital and Yale School of Medicine, New Haven, CT, USA; Genentech, South San Francisco, CA, USA

**Keywords:** marital status, residence characteristics, colonic neoplasms, survival analysis, clinical trial

## Abstract

**Background:**

Limited and conflicting findings have been reported regarding the association between social support and colorectal cancer (CRC) outcomes. We sought to assess the influences of marital status and living arrangement on survival outcomes among patients with stage III colon cancer.

**Patients and Methods:**

We conducted a secondary analysis of 1082 patients with stage III colon cancer prospectively followed in the CALGB 89803 randomized adjuvant chemotherapy trial. Marital status and living arrangement were both self-reported at the time of enrollment as, respectively, married, divorced, separated, widowed, or never-married, and living alone, with a spouse or partner, with other family, in a nursing home, or other.

**Results:**

Over a median follow-up of 7.6 years, divorced/separated/widowed patients experienced worse outcomes relative to those married regarding disease free-survival (DFS) (hazards ratio (HR), 1.44 (95% CI, 1.14-1.81); *P* =.002), recurrence-free survival (RFS) (HR, 1.35 (95% CI, 1.05-1.73); *P = .*02), and overall survival (OS) (HR, 1.40 (95% CI, 1.08-1.82); *P* =.01); outcomes were not significantly different for never-married patients. Compared to patients living with a spouse/partner, those living with other family experienced a DFS of 1.47 (95% CI, 1.02-2.11; *P = .*04), RFS of 1.34 (95% CI, 0.91-1.98; *P = .*14), and OS of 1.50 (95% CI, 1.00-2.25; *P* =.05); patients living alone did not experience significantly different outcomes.

**Conclusion:**

Among patients with stage III colon cancer who received uniform treatment and follow-up within a nationwide randomized clinical trial, being divorced/separated/widowed and living with other family were significantly associated with greater colon cancer mortality. Interventions enhancing social support services may be clinically relevant for this patient population.

**Trial Registration:**

ClinicalTrials.gov Identifier: NCT00003835

## Teaser

Strong social networks have been associated with lower mortality rates. This article assesses the influences of marital status and living arrangement on survival outcomes among stage III colon cancer patients.

**Implications for Practice**

Interventions targeting enhancing social support services for and developing social networks in patients with colon cancer may be an important method by which the significant differences in survival across patients of varying marital statuses and living arrangements can be reduced.

## Introduction

Strong social networks have repeatedly been associated with lower total mortality, and studies examining the association between diminished social networks and mortality risk suggest relative risk estimates comparable to more traditional risk factors, including obesity, cigarette smoking, and hypertension.^1-5^ Marital status in particular has been suggested by some studies to be an independent prognostic factor of survival in patients with cancer, with married patients experiencing superior mortality rates compared with those divorced, separated, widowed, or never-married.^6-13^ Similarly, living arrangement—specifically living alone—has been observed to be associated with inferior cancer survival rates when compared with cohabitating with a spouse, significant other, or partner in some,^14,15^ although not all,^15-20^ studies.

Several studies have previously examined the influence of marital status on colorectal cancer (CRC) outcomes, but findings have been largely inconsistent.^9,13,15,16,21-24^ The majority of prior studies have been limited by their retrospective design and paucity of data on treatment and other confounding factors, rendering it difficult to disentangle the impact of various confounding factors from marital status or living arrangement.

We therefore sought to assess the independent influences of marital status and living arrangement on patient outcomes through secondary analysis of a prospectively-followed cohort nested in a randomized clinical trial (RCT) of adjuvant 5-fluorouracil-based therapy for stage III colon cancer. To our knowledge, this is the first prospective investigation into the impacts of marital status and living arrangement on colon cancer outcomes in North America. We additionally accounted for dietary and lifestyle factors beyond other clinical and sociodemographic variables, thereby conferring a more robust multivariable analysis. Careful and comprehensive documentation during the trial of patient performance status, pathologic stage, post–operative treatment, and diet and lifestyle habits allowed concurrent effects of patient, disease, and treatment characteristics to be examined.

## Patients and Methods

### Study Population

Patients in this RCT were recruited from the US and Canada as participants in the NCI-sponsored Cancer and Leukemia Group B (CALGB; now part of Alliance for Clinical Trials in Oncology) 89803 adjuvant chemotherapy trial for stage III colon cancer, comparing weekly 5-fluorouracil (5-FU) and leucovorin to weekly 5-FU, leucovorin, and irinotecan (ClinicalTrials.gov NCT00003835). One thousand two hundred sixty-four patients were enrolled between April 1999 and April 2001, after the first 87 patients of which the protocol was amended such that patients were required to complete a self-administered questionnaire examining diet and lifestyle behaviors twice: once midway through chemotherapy (approximately 4 months post-surgery; Questionnaire 1), and again 6 months following chemotherapy treatment completion (approximately 14 months post-surgery; Questionnaire 2).

**Figure 1.**
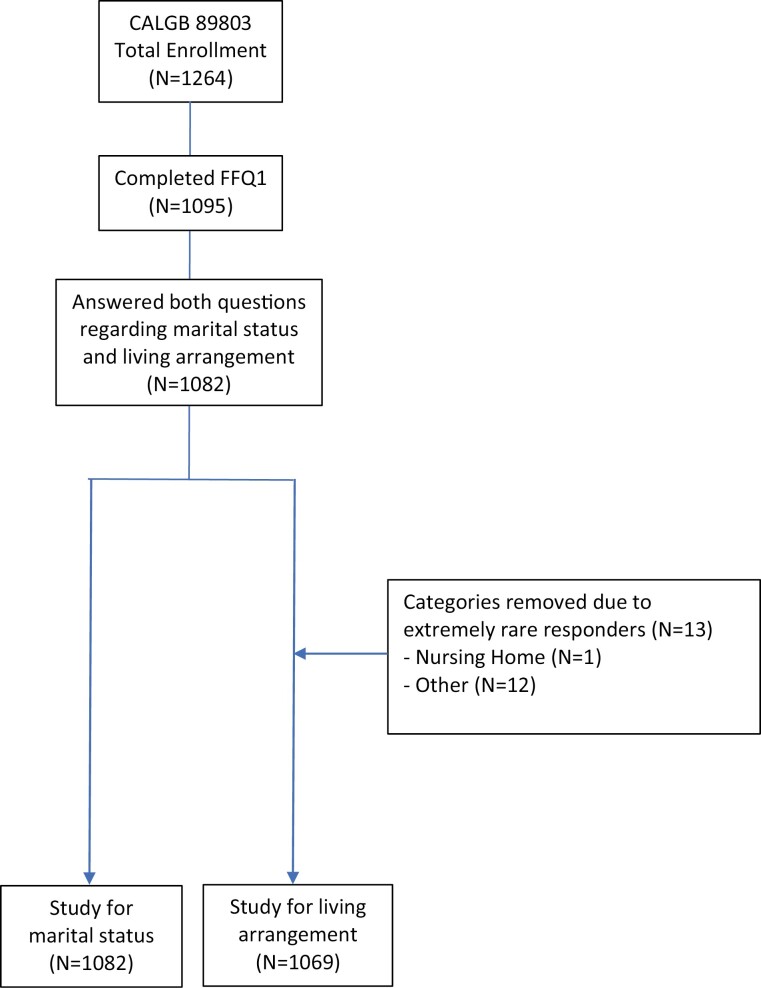
Derivation of the study cohort.

Eligibility required patients to have had a complete surgical resection of the primary tumor within 56 days of trial enrollment, regional lymph node, but no distant, metastases, no prior chemotherapy or radiation treatment for the tumor, a baseline Eastern Cooperative Oncology Group (ECOG) performance status between 0 and 2, and sufficient bone marrow, hepatic, and renal functions. Fig. 1 describes the derivation of the final sample sizes of 1082 and 1069 patients included in this study for marital status and living arrangement analyses, respectively.

### Assessment of Patient Marital Status, Living Arrangement, and Household Income

The marital status and living arrangement of each of the participating subjects were self-reported at the time of enrollment as, respectively, married, divorced, separated, widowed, or never-married, and living alone, with a spouse or partner, with other family, in a nursing home, or other. Analyses by living arrangement were limited to the 1069 subjects who were eligible for CALGB 89803 as described above and whose responses were specified as one of the following: living with spouse/partner, with other family (ie not a spouse/partner), or alone. Those living in a nursing home (*N* = 1) or other situations (*N* = 12) were excluded from the analysis of living arrangement due to the very small number of responders within these categories. Household income was determined by matching the zip codes of patient home addresses, self-reported at the time of enrollment, with publicly available US Census 2000 information.

### Covariate Assessment

Patients completed a validated food frequency questionnaire querying consumption of 131 items over the past 3 months, as previously described.^25-27^ Classification of patients between prudent and Western dietary patterns^28^—characterized by high intakes of fruits and vegetables, poultry, and fish versus high intakes of meat, fat, refined grains, and dessert, respectively—was performed by following the techniques previously described.^29^ Body mass index (BMI), levels of engagement in physical activity, and consistent aspirin use—defined as any aspirin use reported both during (Questionnaire 1) and after completion of adjuvant chemotherapy (Questionnaire 2)—were also recorded. Tumor characteristics were collected via standardized case report forms as part of the treatment trial.^30^

### Endpoints

The primary endpoint for this study was disease-free survival (DFS), defined as the time from study enrollment to recurrence of the tumor, the occurrence of a new primary colon cancer, or death consequent of any cause. Recurrence-free survival (RFS) was defined as the time from study enrollment to recurrence of the tumor, the occurrence of a new primary colon tumor, or death with evidence of recurrence; patients who died with no known tumor recurrence were censored at the last documented evaluation. Overall survival (OS) was defined as the time from study enrollment to death due to any cause.

### Statistical Analysis

Findings from the CALGB 89803 trial for stage III colon cancer comparing adjuvant bolus 5-FU and leucovorin with the combination of bolus 5-FU, leucovorin, and irinotecan have previously been described.^30^ As the 2 chemotherapy treatment arms demonstrated similar results, patient data were combined from both treatment arms and analyzed for this study according to categories of marital status or living arrangement. Baseline characteristics were compared between patients who are married, divorced/separated/widowed, or never-married and patients living with a spouse/partner, alone, or with other family using the Wilcoxon test for continuous variables (age) and Chi-square for the remaining categorical variables.

The Kaplan-Meier method^31^ was performed to estimate the distribution of survival time according to marital status or living arrangement, and the log-rank test was conducted to compare survival between the respective subgroups. Multivariate Cox proportional hazards regression^32^ was used to determine the survival hazard ratios (HR) by marital status (married, divorced/separated/widowed, never-married) or living arrangement (with spouse/partner, alone, with other family), controlling for potential confounders. Three models were built to incrementally examine the association between marital status or living arrangement and the study endpoints. Model 1 was adjusted for age; model 2 was adjusted for age, race, sex, treatment arm, T-stage, number of positive lymph nodes, ECOG performance status, tumor location, presence of clinical bowel obstruction or perforation, consistent aspirin use, insurance status, valid FFQ1, energy intake, BMI, physical activity, Western dietary pattern, and prudent dietary pattern, where the last 5 variables were treated as time-varying covariates in the model as they were collected for and derived from both FFQ; and model 3 was adjusted for all covariates in model 2 in addition to household income, a proxy for socioeconomic status. Missing values for covariates were imputed via the following methods: (1) missing % were less than 5%, and were recoded into the majority category when used as covariates in the Cox model (T-stage, number of positive lymph nodes, performance status, and tumor location); (2) missing % were less than 5%, and were recoded into median values when used as covariates in the Cox model (BMI, physical activity, energy intake, Western dietary pattern, and prudent dietary pattern); (3) missing % was more than 5%, and was recoded as a separate indicator when used as a covariate (household income).

Tests of interaction between marital status or living arrangement and potential confounders were assessed by entering the cross-product of either variable and the covariate of interest. All statistical tests were 2-sided, and *P* values equal to or less than .05 were considered statistically significant. All analyses were conducted using SAS software (version 9.4; SAS Institute, Cary, NC).

Patient registration and clinical data collection were managed and their analyses performed by the Alliance Statistics and Data Center. The statistical analyses were based on the study database frozen on November 9, 2009. Data quality was ensured by review of the data by the Alliance Statistics and Data Center and also by the study chairperson following Alliance policies.

All patients signed study-specific informed consent, which was approved by the NCI Cancer Treatment Evaluation Program and each participating site’s institutional review board.

## Results

### Baseline Characteristics According to Marital Status

Within our cohort, 73.3% of patients self-identified as married, 20.4% as divorced/separated/widowed, and 6.3% as never-married. Table 1 summarizes baseline clinical and sociodemographic characteristics of the study cohort according to marital status. Relative to married patients, divorced/separated/widowed and never-married participants were more likely to be of non-White race, present with a higher T-stage, have a much lower or higher BMI, and engage in less physical activity. Divorced/separated/widowed patients were additionally less likely to be male and have private or no insurance over public insurance, and those who had never been married were more likely to be younger.

**Table 1.** Baseline characteristics of 1082 patients with stage III colon cancer by marital status.

	Married	Divorced/separated/widowed	Never-married	*P-*value∗
# Deaths or recurrence/at risk	#302/793	#108/221	#24/68	
Age, median(Q1-Q3)	60.0 (52.0-69.0)	64.0 (55.0-70.0)	52.0 (43.5-61.5)	<.001
Age, years, no. (%)				
≤60	408 (51.5%)	94 (42.5%)	50 (73.5%)	<.001
>60	385 (48.5%)	127 (57.5%)	18 (26.5%)	
Sex, no. (%)				
Male	475 (59.9%)	89 (40.3%)	41 (60.3%)	<.001
Female	318 (40.1%)	132 (59.7%)	27 (39.7%)	
Race, no. (%)				
White	728 (91.8%)	170 (76.9%)	57 (83.8%)	<.001
Other	65 (8.2%)	51 (23.1%)	11 (16.2%)	
Treatment arm, no. (%)				
5-FU/LV	397 (50.1%)	119 (53.8%)	33 (48.5%)	.57
IFL	396 (49.9%)	102 (46.2%)	35 (51.5%)	
T-stage, no. (%)^a^				
Missing	17 (2.1%)	6 (2.7%)	3 (4.4%)	.05
T1-2	114 (14.4%)	20 (9.1%)	5 (7.4%)	
T3-4	662 (83.5%)	195 (88.2%)	60 (88.2%)	
Number of positive lymph nodes, no. (%)				
Missing	13 (1.6%)	5 (2.3%)	3 (4.4%)	.19
1-3	501 (63.2%)	128 (57.9%)	46 (67.7%)	
4+	279 (35.2%)	88 (39.8%)	19 (27.9%)	
Performance status, no. (%)^b^				
Missing	15 (1.9%)	6 (2.7%)	3 (4.4%)	.26
ECOG 0	594 (74.9%)	154 (69.7%)	46 (67.7%)	
ECOG 1,2	184 (23.2%)	61 (27.6%)	19 (27.9%)	
Clinical bowel obstruction or perforation, no. (%)				
No	613 (77.3%)	179 (81.0%)	50 (73.5%)	.34
Yes	180 (22.7%)	42 (19.0%)	18 (26.5%)	
Tumor location, no. (%)				
Missing	15 (1.9%)	7 (3.2%)	3 (4.4%)	.02
Distal	352 (44.4%)	74 (33.5%)	28 (41.2%)	
Proximal	426 (53.7%)	140 (63.4%)	37 (54.4%)	
Valid FFQ1, no. (%)				
*** *** No	18 (2.3%)	15 (6.8%)	5 (7.4%)	.001
*** *** Yes	775 (97.7%)	206 (93.2%)	63 (92.7%)	
Consistent aspirin use (reported use in both FFQ 1&2), no. (%)				
No	727 (91.7%)	206 (93.2%)	65 (95.6%)	.43
Yes	66 (8.3%)	15 (6.8%)	3 (4.4%)	
BMI, no. (%)				
Missing	18 (2.3%)	15 (6.8%)	5 (7.4%)	.05
Min-24.9	249 (31.4%)	73 (33.0%)	24 (35.3%)	
25-29.9	302 (38.1%)	60 (27.2%)	17 (25.0%)	
30-max	224 (28.3%)	73 (33.0%)	22 (32.4%)	
Physical activity, no. (%)				
Missing	23 (2.9%)	17 (7.7%)	5 (7.4%)	.02
0-2.9 met-h	283 (35.7%)	96 (43.4%)	28 (41.2%)	
3 met-h+	487 (61.4%)	108 (48.9%)	35 (51.5%)	
Energy intake, no. (%)^c^				
Missing	18 (2.3%)	15 (6.8%)	5 (7.4%)	.27
<Median	387 (48.8%)	109 (49.3%)	26 (38.2%)	
≥Median	388 (48.9%)	97 (43.9%)	37 (54.4%)	
Western dietary pattern, no. (%)^c^				
Missing	18 (2.3%)	15 (6.8%)	5 (7.4%)	.22
<Median	376 (47.4%)	114 (51.6%)	32 (47.1%)	
≥Median	399 (50.3%)	92 (61.6%)	31 (45.6%)	
Prudent dietary pattern, no. (%)^c^				
Missing	18 (2.3%)	15 (6.8%)	5 (7.4%)	.65
<Median	381 (48.1%)	108 (48.9%)	33 (48.5%)	
≥Median	394 (49.7%)	98 (44.3%)	30 (44.1%)	
Household income, no. (%)^c^				
Missing	196 (24.7%)	42 (19.0%)	17 (25.0%)	.17
<Median	286 (36.1%)	99 (44.8%)	28 (41.2%)	
≥Median	311 (39.2%)	80 (36.2%)	23 (33.8%)	
Insurance status, no. (%)				
Self-pay/private	527 (66.5%)	125 (56.6%)	47 (69.1%)	.02
Medicare/Medicaid/Military/Other	266 (33.5%)	96 (43.4%)	21 (30.9%)	

T1-2, level of invasion through the bowel wall not beyond the muscle layer; T3-4, level of invasion through the bowel wall beyond the muscle layer.

Baseline performance status: Performance status 0 = fully active; performance status 1 = restricted in physically strenuous activity but ambulatory and able to carry out light work; performance status 2 = ambulatory and capable of all self-care but unable to carry out any work activities, up and about more than 50% of waking hours.

Median among all 1082 patients for each factor.

*P*-value based on^1^ Wilcoxon test for continuous variable (age); or^2^ Chi-square test for categorical variables.

Abbreviations: 5-FU, 5-fluorouracil; LV, leucovorin; IFL, irinotecan, 5-fluorouracil, leucovorin; FFQ, food frequency questionnaire; BMI, body mass index.

### Association Between Marital Status and Cancer Recurrence or Mortality

**Figure 2.**
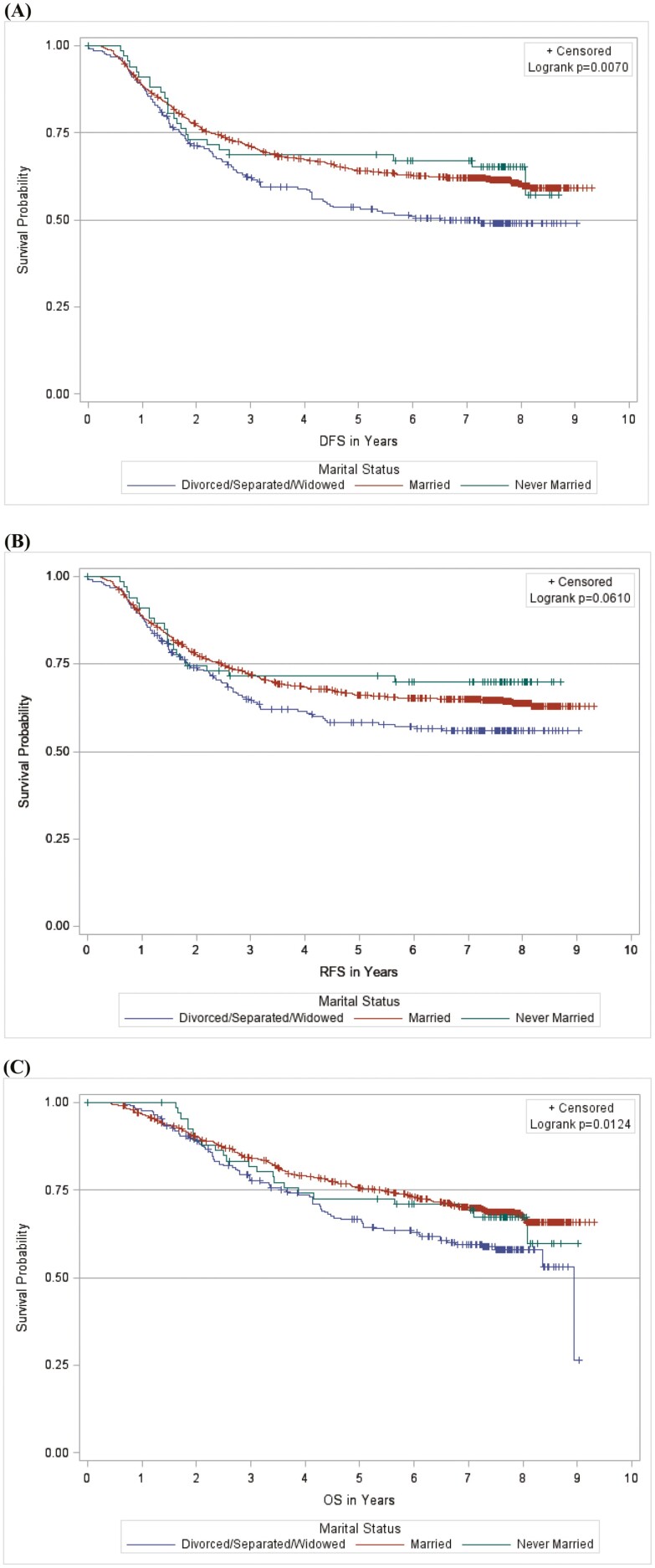
Kaplan-Meier curves according to marital status for (A) disease-free survival, (B) recurrence-free survival, and (C) overall survival.

Over a median follow-up of 7.6 years, there were 434, 388, and 348 events for DFS, RFS, and OS analyses, respectively. In a Kaplan-Meier analysis, divorced/separated/widowed patients experienced an inferior DFS (log-rank *P* = .01) and OS (log-rank *P* = .01) relative to married and never-married patients (Fig. 2). In multivariable Cox regression analyses (Table 2), divorced/separated/widowed patients experienced worse adjusted hazard ratios (HRs) of 1.44 (95% CI, 1.14-1.81; *P* = .002) for DFS, 1.35 (95% CI, 1.05-1.73; *P*=.02) for RFS, and 1.40 (95% CI, 1.08-1.82; *P* =.01) for OS, when compared with married subjects. Never-married patients experienced similar survival rates relative to those married with a DFS of 1.00 (95% CI, 0.66-1.53; *P* = .99), RFS of 0.88 (95% CI, 0.55-1.40; *P* =.59), and OS of 1.22 (95% CI, 0.78-1.91; *P* =.39).

**Table 2.** Marital status, colon cancer recurrence, and mortality.

	Married	Divorced/separated/widowed		Never-married	
		HR	*P*-value	HR	*P*-value
Disease-free survival					
# Event/at risk	#302/793	#108/221		#24/68	
Age-adjusted only, HR (95% CI)	Ref	1.38 (1.10-1.72)	0.005	0.97 (0.64-1.47)	.88
Multivariable adjusted, HR (95% CI)^a^	Ref	1.44 (1.14-1.81)	0.002	0.99 (0.65-1.52)	.97
Multivariable adjusted, HR (95% CI)^b^	Ref	1.44 (1.14-1.81)	0.002	1.00 (0.66-1.53)	.99
Recurrence-free survival					
# Event/At risk	#276/793	#92/221		#20/68	
Age-adjusted only, HR (95% CI)	Ref	1.29 (1.02-1.63)	0.04	0.85 (0.54-1.35)	.49
Multivariable adjusted, HR (95% CI)^a^	Ref	1.35 (1.05-1.72)	0.02	0.87 (0.55-1.38)	.56
Multivariable adjusted, HR (95% CI)^b^	Ref	1.35 (1.05-1.73)	0.02	0.88 (0.55-1.40)	.59
Overall survival					
# Event/At risk	#239/793	#87/221		#22/68	
Age-adjusted only, HR (95% CI)	Ref	1.38 (1.08-1.77)	0.01	1.21 (0.78—1.88)	.40
Multivariable adjusted, HR (95% CI)^a^	Ref	1.40 (1.08-1.82)	.01	1.21 (0.77-1.89)	.41
Multivariable adjusted, HR (95% CI)^b^	Ref	1.40 (1.08-1.82)	.01	1.22 (0.78-1.91)	.39

A chi-square goodness of fit test with *P* < .001 suggested significant difference between 3 levels of marital status.

Multivariable-adjusted model adjusted for age (continuous), sex (male, female), race (White, other), treatment arm, T-stage (T1-2, T3-4), number of positive nodes (1-3, 4+), performance status (ECOG 0, ECOG 1-2), tumor location (proximal, distal), clinical bowel obstruction or perforation (yes, no), consistent aspirin use (yes, no), insurance status (self-pay/private, Medicare/Medicaid/Military/Other), valid FFQ1 (yes, no), time-varying energy intake, BMI, physical activity, Western dietary pattern, prudent dietary pattern (all time-varying variables are continuous).

Multivariable-adjusted model adjusted for age (continuous), sex (male, female), race (White, other), treatment arm, T-stage (T1-2, T3-4), number of positive nodes (1-3, 4+), performance status (ECOG 0, ECOG 1-2), tumor location (proximal, distal), clinical bowel obstruction or perforation (yes, no), consistent aspirin use (yes, no), insurance status (self-pay/private, Medicare/Medicaid/Military/Other), valid FFQ1 (yes, no), household income, time-varying energy intake, BMI, physical activity, Western dietary pattern, prudent dietary pattern (all time-varying variables are continuous).

### Stratified Analyses of Marital Status by Potential Effect Modifiers

We examined the influence of marital status on DFS and OS across strata of other potential predictors of patient outcome (Supplementary Tables 1 and 2). The association between marital status and patient outcome was not significantly modified across all examined strata of patient, disease, and treatment characteristics. However, in these stratified analyses, statistical power to adequately detect differences was limited by the sample size, and such analyses should be considered exploratory.

### Baseline Characteristics According to Living Arrangement

Within our cohort, 76.4% of patients self-identified as living with a spouse/partner, 16.0% as living alone, and 7.6% as living with other family besides a spouse/partner. Relative to patients living with a spouse/partner, those living alone or with other family were more likely to be female, present with a higher T-stage, have a more proximal tumor, and engage in less physical activity (Table 3). Patients living with other family were also more likely to be older and of non-White race, while patients living alone were more likely to have a lower BMI.

**Table 3.** Baseline characteristics of 1069 patients with stage III colon cancer by living arrangement.

	With spouse/partner	Alone	With other family	*P*-value
# Deaths or recurrence/at risk	#315/817	#76/171	#36/81	
Age, median (Q1-Q3)	60 (51-59)	64 (55-70)	56 (48-67)	.0002
Age, years, no. (%)				
≤60	422 (51.7%)	71 (41.5%)	52 (64.2%)	.003
>60	395 (48.3%)	100 (58.5%)	29 (35.8%)	
Sex, no. (%)				
Male	498 (61.0%)	68 (39.8%)	29 (35.8%)	<.00010
Female	319 (39.0%)	103 (60.2%)	52 (64.2%)	
Race, no. (%)				
White	750 (91.8%)	139 (81.3%)	55 (67.9%)	<.00010
Other	67 (8.2%)	32 (18.7%)	26 (32.1%)	
Treatment arm, no. (%)				
5-FU/LV	412 (50.4%)	85 (49.7%)	45 (55.6%)	.65
IFL	405 (49.6%)	86 (50.3%)	36 (44.4%)	
T-stage, no. (%)^a^				
Missing	19 (2.3%)	1 (0.58%)	5 (6.2%)	.03
T1-2	117 (14.3%)	13 (7.6%)	7 (8.6%)	
T3-4	681 (83.4%)	157 (91.8%)	69 (85.2%)	
Number of positive lymph nodes, no. (%)				
Missing	15 (1.8%)	1 (0.58%)	4 (4.9%)	.53
1-3	514 (62.9%)	103 (60.2%)	52 (64.2%)	
4+	288 (35.3%)	67 (39.2%)	25 (30.9%)	
Performance status, no. (%)^b^				
Missing	17 (2.1%)	1 (0.58%)	5 (6.2%)	.07
ECOG 0	614 (75.2%)	123 (71.9%)	50 (61.7%)	
ECOG 1,2	186 (22.8%)	47 (27.5%)	26 (32.1%)	
Clinical bowel obstruction or perforation, no. (%)				
No	630 (77.1%)	139 (81.3%)	62 (76.5%)	.47
Yes	187 (22.9%)	32 (18.7%)	19 (23.5%)	
Tumor location, no. (%)				
Missing	18 (2.2%)	2 (1.2%)	4 (4.9%)	.008
Distal	365 (44.7%)	57 (33.3%)	28 (34.6%)	
Proximal	434 (53.1%)	112 (65.5%)	49 (60.5%)	
Consistent aspirin use (both FFQ1&2), no. (%)				
No	753 (92.2%)	161 (94.2%)	72 (88.9%)	.34
Yes	64 (7.8%)	10 (5.8%)	9 (11.1%)	
BMI, no. (%)				
Missing	20 (2.5%)	9 (5.3%)	8 (9.8%)	.02
Min-24.9	256 (31.3%)	64 (37.4%)	21 (25.9%)	
25-29.9	308 (37.7%)	47 (27.5%)	21 (25.9%)	
30-max	233 (28.5%)	51 (29.8%)	31 (38.3%)	
Physical activity, no. (%)				
Missing	25 (3.1%)	11 (6.4%)	8 (9.9%)	.004
0-2.9 met-h	290 (35.5%)	75 (43.9%)	38 (46.9%)	
3 met-h+	502 (61.4%)	85 (49.7%)	35 (43.2%)	
Energy intake, no. (%)				
Missing	20 (2.5%)	9 (5.3%)	8 (9.9%)	.51
<Median	393 (48.1%)	88 (51.5%)	37 (45.7%)	
≥Median	404 (49.5%)	74 (43.3%)	36 (44.4%)	
Western dietary pattern, no. (%)				
Missing	20 (2.5%)	9 (5.3%)	8 (9.9%)	.26
<Median	389 (47.6%)	89 (542.1%)	40 (49.4%)	
≥Median	408 (49.9%)	73 (42.7%)	33 (40.7%)	
Prudent dietary pattern, no. (%)				
Missing	20 (2.5%)	9 (5.3%)	8 (9.9%)	.92
<Median	395 (48.4%)	83 (48.5%)	36 (44.4%)	
≥Median	402 (49.2%)	79 (46.2%)	37 (45.7%)	
Household income, no. (%)				
Missing	203 (24.9%)	35 (20.5%)	12 (14.8%)	.07
<Median	292 (35.7%)	73 (42.7%)	42 (51.9%)	
≥Median	322 (39.4%)	63 (36.8%)	27 (33.3%)	
Insurance status, no. (%)				
Self-pay/private	539 (66.0%)	99 (57.9%)	54 (66.7%)	.12
Medicare/Medicaid/Military/Other	278 (34.0%)	72 (42.1%)	27 (33.3%)	

Abbreviations: 5-FU = 5-fluorouracil; LV = leucovorin; IFL = irinotecan, 5-fluorouracil, leucovorin; FFQ = food frequency questionnaire; BMI = body mass index.

T1-2 = level of invasion through the bowel wall not beyond the muscle layer; T3-4 = level of invasion through the bowel wall beyond the muscle layer.

Baseline performance status: performance status 0 = fully active; performance status 1 = restricted in physically strenuous activity but ambulatory and able to carry out light work; performance status 2 = ambulatory and capable of all self-care but unable to carry out any work activities, up and about more than 50% of waking hours.

*P*-value based on (1) Wilcoxon test for continuous variable (age); or (2) Chi-square test for categorical variables.

### Impact of Living Arrangement on Cancer Recurrence or Mortality

**Figure 3.**
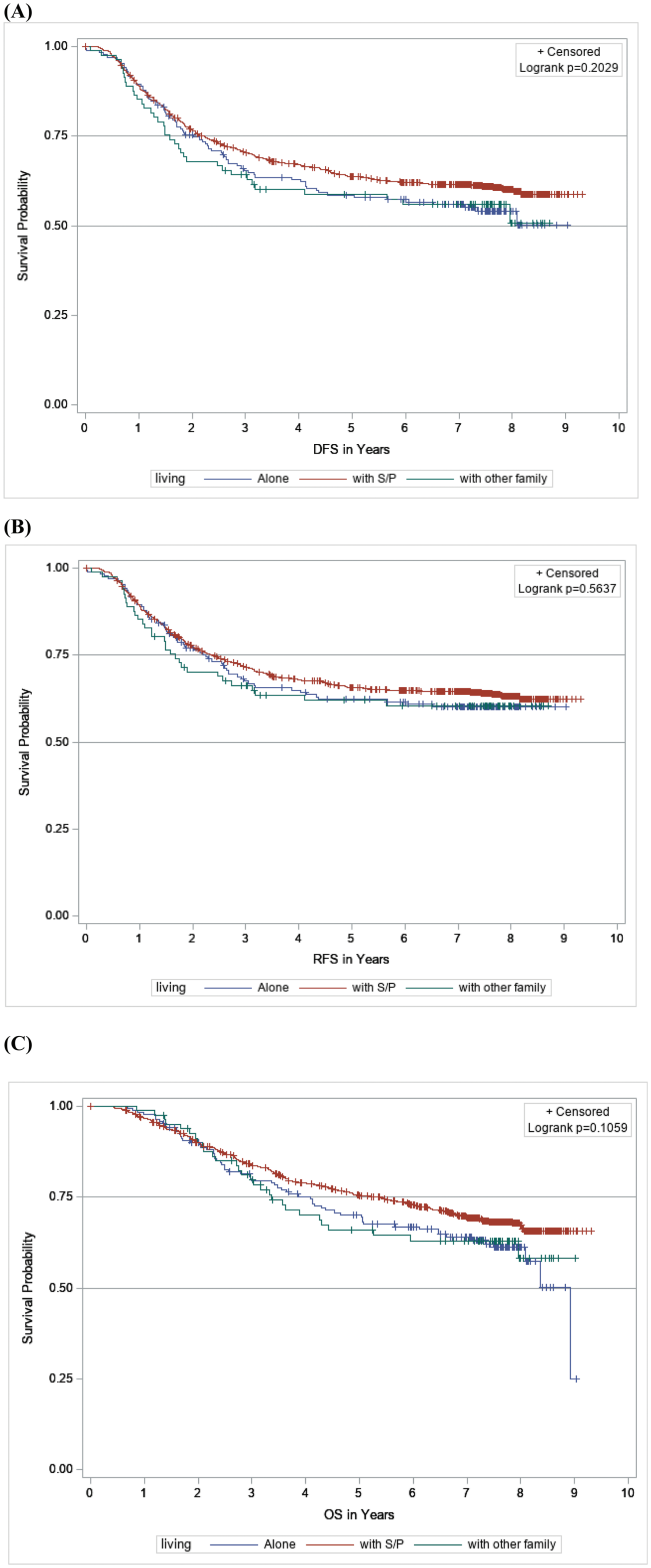
Kaplan-Meier curves according to living arrangement for (A) disease-free survival, (B) recurrence-free survival, and (C) overall survival.

In the cohort of 1069 participants with living arrangement data, there were 427, 384, and 341 events for DFS, RFS, and OS analyses, respectively. In a Kaplan-Meier analysis, no significant differences were found in DFS (log-rank *P* =.20) or OS (log-rank *P* =.11) by living arrangement (Fig. 3). In multivariable Cox regression analyses, patients living with other family experienced inferior DFS and OS when compared with those living with a spouse/partner (Table 4). The fully adjusted HRs for patients living with other family were 1.47 (95% CI, 1.02-2.11; *P* = .04) for DFS, 1.34 (95% CI, 0.91-1.98; *P* =.14) for RFS, and 1.50 (95% CI, 1.00-2.25; *P* =.05) for OS, when compared with patients living with a spouse/partner. Patients living alone experienced non-significant worse survival relative to those living with a spouse/partner with a DFS of 1.24 (95%CI, 0.96-1.61; *P* =.11), RFS of 1.14 (95%CI, 0.86-1.51; *P* =.35), and OS of 1.30 (95% CI, 0.98-1.73; *P* =.07).

**Table 4.** Living arrangement, colon cancer recurrence, and mortality.

	With spouse/partner	Alone		Other family	
		HR	*P*-value	HR	*P*-value
Disease-free survival					
# Event/at risk	#315/817	#76/171		#36/81	
Age-adjusted only, HR (95% CI)	Ref	1.17 (0.91-1.51)	.22	1.29 (0.91-1.82)	.15
Multivariable adjusted, HR (95% CI)^a^	Ref	1.24 (0.96-1.61)	.10	1.45 (1.01-2.08)	.05
Multivariable adjusted, HR (95% CI)^b^	Ref	1.24 (0.96-1.61)	.11	1.47 (1.02-2.11)	.04
Recurrence-free survival					
# Event/at risk	#288/817	#65/171		#31/81	
Age-adjusted only, HR (95% CI)	Ref	1.11 (0.84-1.45)	.46	1.18 (0.81-1.71)	.38
Multivariable adjusted, HR (95% CI)^a^	Ref	1.14 (0.87-1.51)	.34	1.32 (0.90-1.94)	.16
Multivariable adjusted, HR (95% CI)^b^	Ref	1.14 (0.86-1.51)	.35	1.34 (0.91-1.98)	.14
Overall survival					
# Event/at risk	#248/817	#64/171		#29/81	
Age-adjusted only, HR (95% CI)	Ref	1.25 (0.95-1.64)	.12	1.35 (0.92-1.99)	.13
Multivariable adjusted, HR (95% CI)^a^	Ref	1.31 (0.98-1.74)	.06	1.47 (0.98-2.21)	.06
Multivariable adjusted, HR (95% CI)^b^	Ref	1.30 (0.98-1.73)	.07	1.50 (1.00-2.25)	.05

A chi-square goodness of fit test with *P* <.001 suggested significant difference between 3 levels of living arrangement.

Multivariable-adjusted model adjusted for age (continuous), sex (male, female), race (White, other), treatment arm, T-stage (T1-2, T3-4), number of positive nodes (1-3, 4+), performance status (ECOG 0, ECOG 1-2), tumor location (proximal, distal), clinical bowel obstruction or perforation (yes, no), consistent aspirin use (yes, no), insurance status (self-pay/private, Medicare/Medicaid/Military/Other), valid FFQ1 (yes, no), time-varying energy intake, BMI, physical activity, Western dietary pattern, prudent dietary pattern (all time-varying variables are continuous).

Multivariable-adjusted model adjusted for age (continuous), sex (male, female), race (White, other), treatment arm, T-stage (T1-2, T3-4), number of positive nodes (1-3, 4+), performance status (ECOG 0, ECOG 1-2), tumor location (proximal, distal), clinical bowel obstruction or perforation (yes, no), consistent aspirin use (yes, no), insurance status (self-pay/private, Medicare/Medicaid/Military/other), valid FFQ1 (yes, no), household income, time-varying energy intake, BMI, physical activity, Western dietary pattern, prudent dietary pattern (all time-varying variables are continuous).

### Stratified Analyses of Living Arrangement by Potential Effect Modifiers

We further examined whether the influence of living arrangement on DFS and OS differed across strata of other potential predictors of patient outcome (Supplementary Tables 3 and 4). The association between living arrangement and patient outcome was not significantly modified across all examined strata of patient, disease, and treatment characteristics. As previously mentioned, in these stratified analyses, statistical power to adequately detect differences was limited by the sample size, and such analyses should be considered exploratory.

## Discussion

In this secondary analysis of prospectively-followed, resected patients with stage III colon cancer enrolled in a post–operative adjuvant chemotherapy clinical trial, we found divorced/separated/widowed patients experienced significantly worse DFS, RFS, and OS compared to those married, and patients living with other family experienced decreased DFS and OS relative to those living with a spouse/partner. The inferior patient outcomes associated with being divorced/separated/widowed and living with other family remained consistent after adjusting for multiple predictors of patient outcome and across strata of patient, disease, and treatment characteristics, including household income and dietary and lifestyle factors. Our study is, to our knowledge, the first North American prospective investigation into the associations between marital status and living arrangement and colon cancer patient outcomes.

A limited number of studies have examined these factors in patients with CRC. A few studies have reported worse cancer-specific mortality in non-married CRC patients relative to those married^16,21^ or among CRC patients living alone compared to those living with someone.^14,15^ In contrast, other studies have reported mixed or non-significant associations between marital status and CRC outcomes.^15,22,24^ These studies were, however, mostly conducted with northern European patient populations, and international differences in cultural and social dynamics between current and past spouses/partners may have resulted in these discrepant findings. Indeed, a recent US-based SEER investigation showed that cancer-specific survival has drastically improved since 1990 for patients of all marital statuses except for those widowed.^23^ Studies of patients diagnosed with malignancies other than CRC have also reported inferior outcomes among divorced, separated, and widowed patients when compared with those married or never-married.^12,17,18,33,34^

It is important to note the growing body of literature suggesting an association between greater allostatic burden—the cumulative biological burden of chronic stress experienced during life—and worse health outcomes via dysregulation of cardiovascular, immune, and metabolic systems.^35,36^ In the context of cancer, chronic stress may result in impaired tumor immune surveillance^37^ or DNA damage^38^; indeed, a higher allostatic load has been associated with increased overall cancer-specific mortality.^39,40^ Among patients with breast cancer, it has recently also been correlated with worse chemotherapy completion rates.^41^ Although no studies to date have examined the role of allostatic load in CRC outcomes, our finding that disruptions in or loss of relationships—as is the case in divorce, separation, and widowhood—are associated with worse survival, rather than never having been married, may be supportive of this notion. Future research should additionally address the role of allostatic load in influencing patient outcomes.

With respect to living arrangement, we found that patients residing with other family experienced inferior outcomes when compared with those living with a spouse or partner. Although patients in our cohort who were living with other family were more likely to be obese, engage in less physical activity, present with a worse performances status, and possess a lower household income, our results remained largely unchanged, even after adjusting for these factors. Despite adjusting for these variables in our analyses, residual confounding or additional confounding by comorbidities may have contributed to the observed worse outcomes in this group. The meaning of this finding is ultimately less clear, but may be attributable to the other family members these patients were living with potentially being dependents whose care or rearing the patients may have prioritized over their own recovery.

Our overall findings lend support for the general hypothesis that stronger social networks are associated with improved outcomes among colon cancer patients. A variety of mechanisms have been proposed to contribute to the better outcomes observed in cancer patients with greater social support. Married patients are more likely to present at an earlier stage, undergo more definitive treatment, demonstrate greater treatment adherence, and receive any treatment at all,^9,11,16^ potentially due to the behavior- and health-monitoring influences conferred by having a spouse.^42,43^

Social networks and living situations can influence dietary and lifestyle factors, and such behaviors have long been demonstrated to influence both CRC risk and outcomes.^44-46^ However, in the current study, we observed significant associations between both marital status and living arrangement and patient outcome even after adjusting for prospectively-collected data on dietary and lifestyle factors as well as median household income. As the nature and quality of treatment were standardized across our entire cohort of all same-stage patients, our results offer some evidence for the notion that psychosocial support garnered through social networks can influence cancer patient outcomes.

Assessing relationships between marital status or living arrangement and patient outcomes in colon cancer through an RCT offers several strengths. By studying patients enrolled in a clinical trial, we potentially reduced the biases introduced by differences in access to healthcare resources unavoidable in population-based cancer registries. Moreover, as all patients in this study met the same enrollment criteria and received adjuvant 5-FU-based chemotherapy, confounding by patient characteristics or the nature of therapy was minimized. Finally, all patients had stage III colon cancer, minimizing the effect of disease stage heterogeneity on outcomes.

Our study is not without limitations. Patients who choose to enroll in clinical trials may differ from the general population: they must meet specific eligibility criteria, be chosen as appropriate candidates, and have the motivation to participate. Nonetheless, the overall outcomes for patients in this trial were comparable to those of a similarly staged population in the SEER database. Moreover, CALGB 89803 enrolled patients from both community and academic centers across North America, thereby lowering the likelihood of biased sampling, and the cohort appears to have characteristics representative of the larger population of patients with stage III colon cancer. Marital status and living arrangement were also noted only at the time of trial enrollment. Although any changes during the trial or follow-up period would therefore have been missed, incorporation of such changes—at least with respect to marital status—has been shown in some cases to paradoxically decrease the observed benefits of marriage.^47^ Furthermore, household income was determined indirectly using zip codes and publicly available US census data as a proxy. Despite these values thus reflecting the median income of each patient’s neighborhood, this is a well-established methodology and unlikely to have significantly confounded our results. The presence of residual confounding cannot be excluded; however, our findings remained consistent even after controlling for both known and suspected patient outcome predictors. Additional confounding by comorbidities may have still contributed to the observed worse outcomes among patients residing with other family or those who were divorced/separated/widowed, although enrollment into this RCT was restricted to patients with a relatively normal baseline ECOG performance status. Lastly, the data collection for the original RCT was performed during a time when marriage was not available to same-sex couples living in the US, and our findings relating to marital status may thus not be generalizable to individuals in non-heterosexual relationships.

## Conclusion

In conclusion, we found being divorced/separated/widowed or living with other family were significantly associated with worse colon cancer recurrence and mortality—relative to being married or living with a spouse/partner, respectively—in this cohort of patients with stage III colon cancer treated within an RCT. National US census data from the 2018 Current Population Survey Annual Social and Economic Supplement^48^ indicate temporal trends of increasingly delayed age at first marriage and greater prevalence of single-person households. Americans are also now almost 3 times more likely than in the past to report having no one in their lives with whom they can discuss serious matters.^49^ Interventions targeting enhancing social support services and developing social networks in patients with colon cancer may be an important method by which the significant differences in survival across patients of varying marital statuses and living arrangements can be reduced.

## Supplementary Material

oyab070_suppl_Supplementary_Tables

## Data Availability

The data underlying this article will be shared on reasonable request to the corresponding author.
