# Supplementary material for: Marital Status, Living Arrangement, and Cancer Recurrence and Survival in Patients with Stage III Colon Cancer: Findings from CALGB 89803 (Alliance)
Source: Oncologist. 2022 Feb 19;27(6):e494–505. doi: 10.1093/oncolo/oyab070 (PMC9177101; doi:10.1093/oncolo/oyab070)
Supplement: oyab070_suppl_Supplementary_Tables [file oyab070_suppl_supplementary_tables.docx]

**Supplementary Table 1.** Disease-free survival by marital status stratified by clinicopathological and FFQ features.^a^

| **Subgroup** | **Count (%)** | **Married – HR** | **D/S/W – HR** | **D/S/W – *P-*value** | **Never-Married – HR** | **Never-Married – *P-*value** | ***P*_interaction_^b^** |
| --- | --- | --- | --- | --- | --- | --- | --- |
| ***Age (years)*** |  |  |  |  |  |  | 0.42 |
| ≤60 | 552(51.0) | ref | 1.71(1.22-2.41) | 0.002 | 1.08(0.65-1.79) | 0.78 |  |
| >60 | 530(49.0) | ref | 1.28(0.94-1.73) | 0.11 | 0.86(0.40-1.85) | 0.71 |  |
| ***Sex*** |  |  |  |  |  |  | 0.13 |
| Male | 605(55.9) | ref | 1.30(0.94-1.80) | 0.11 | 1.35(0.82-2.22) | 0.24 |  |
| Female | 477(44.1) | ref | 1.58(1.14-2.20) | 0.007 | 0.58(0.25-1.32) | 0.19 |  |
| ***Treatment arm*** |  |  |  |  |  |  | 0.99 |
| 5-FU/LV | 549(50.7) | ref | 1.45(1.06-1.97) | 0.02 | 0.98(0.53-1.82) | 0.95 |  |
| IFL | 533(49.3) | ref | 1.43(1.02-1.99) | 0.04 | 1.02(0.57-1.82) | 0.94 |  |
| ***T-stage*** |  |  |  |  |  |  | 0.99 |
| T1-2 ^d^ | 139(13.2) | ref | 1.54(0.67-3.54) | 0.31 | -- | -- |  |
| T3-4 | 917(86.8) | ref | 1.43(1.12-1.82) | 0.004 | 1.11(0.72-1.70) | 0.64 |  |
| ***Number of positive lymph nodes*** |  |  |  |  |  |  | 0.82 |
| 1-3 | 675(63.6) | ref | 1.37(0.99-1.90) | 0.06 | 0.96(0.56-1.65) | 0.88 |  |
| 4+ | 386(36.4) | ref | 1.51(1.10-2.09) | 0.01 | 1.21(0.61-2.41) | 0.58 |  |
| ***Performance status ^c^*** |  |  |  |  |  |  | 0.51 |
| ECOG0 | 794(75.0) | ref | 1.44(1.09-1.89) | 0.01 | 1.24(0.76-2.02) | 0.4 |  |
| ECOG1,2 | 264(25.0) | ref | 1.51(1.00-2.28) | 0.05 | 0.72(0.31-1.66) | 0.43 |  |
| ***Clinical bowel obstruction or perforation*** |  |  |  |  |  |  | 0.59 |
| No | 842(77.8) | ref | 1.38(1.06-1.79) | 0.02 | 0.89(0.53-1.50) | 0.67 |  |
| Yes | 240(22.2) | ref | 1.66(1.04-2.65) | 0.03 | 1.32(0.63-2.79) | 0.47 |  |
| ***Tumor location*** |  |  |  |  |  |  | 0.92 |
| Distal | 454(43.0) | ref | 1.50(1.03-2.18) | 0.04 | 0.98(0.51-1.89) | 0.95 |  |
| Proximal | 603(57.0) | ref | 1.40(1.05-1.88) | 0.02 | 1.10(0.63-1.92) | 0.75 |  |
| ***BMI*** |  |  |  |  |  |  | 0.3 |
| Min-24.9 | 346(33.1) | ref | 1.18(0.79-1.76) | 0.41 | 1.15(0.60-2.22) | 0.67 |  |
| 25-29.9 | 379(36.3) | ref | 1.98(1.32-2.96) | 0.0009 | 0.96(0.39-2.39) | 0.93 |  |
| 30-max | 319(30.6) | ref | 1.28(0.86-1.90) | 0.23 | 0.70(0.32-1.52) | 0.36 |  |
| ***Physical activity*** |  |  |  |  |  |  | 0.51 |
| 0-2.9 met-h | 407(39.2) | ref | 1.31(0.92-1.85) | 0.13 | 0.73(0.37-1.46) | 0.38 |  |
| 3 met-h+ | 630(60.8) | ref | 1.55(1.13-2.12) | 0.007 | 1.15(0.65-2.05) | 0.62 |  |
| ***Western dietary pattern*** |  |  |  |  |  |  | 0.34 |
| <Median | 522(50.0) | ref | 1.53(1.11-2.12) | 0.01 | 0.68(0.34-1.33) | 0.26 |  |
| ≥Median | 522(50.0) | ref | 1.35(0.96-1.89) | 0.08 | 1.22(0.68-2.18) | 0.51 |  |
| ***Prudent dietary pattern*** |  |  |  |  |  |  | 0.37 |
| <Median | 522(50.0) | ref | 1.46(1.08-1.98) | 0.02 | 1.22(0.71-2.10) | 0.46 |  |
| ≥Median | 522(50.0) | ref | 1.37(0.96-1.96) | 0.08 | 0.63(0.29-1.35) | 0.23 |  |
| ***Household income*** |  |  |  |  |  |  | 0.4 |
| <Median | 413(49.9) | ref | 1.66(1.15-2.41) | 0.007 | 1.17(0.61-2.23) | 0.63 |  |
| ≥Median | 414(50.1) | ref | 1.20(0.83-1.74) | 0.34 | 0.81(0.37-1.77) | 0.6 |  |
| ***Insurance Status*** |  |  |  |  |  |  | 0.78 |
| Self-Pay/Private | 699(64.6) | ref | 1.53(1.12-2.08) | 0.007 | 1.08(0.64-1.82) | 0.78 |  |
| Medicare/Medicaid/Military/Other | 383(35.4) | ref | 1.33(0.95-1.87) | 0.1 | 0.88(0.43-1.81) | 0.73 |  |

Abbreviations: 5-FU = 5-fluorouracil; LV = leucovorin; IFL = irinotecan, 5-fluorouracil, leucovorin; FFQ = food frequency questionnaire; BMI = body mass index

^a^ Multivariable-adjusted model adjusted for age (continuous), sex (male, female), race (White, other), treatment arm, T-stage (T1-2, T3-4), number of positive nodes (1-3, 4+), performance status (ECOG 0, ECOG 1-2), tumor location (proximal, distal), clinical bowel obstruction or perforation (yes, no), consistent aspirin use (yes, no), insurance status (Self-Pay/Private, Medicare/Medicaid/Military/Other), valid FFQ1 (yes, no), household income, time-varying energy intake, BMI, physical activity, Western dietary pattern, prudent dietary pattern (all time-varying variables are continuous).

^b^ Interaction term built as a cross-product of marital status and the covariate of interest as binary variables.

^c^ Baseline performance status: Performance status 0 = fully active; Performance status 1 = restricted in physically strenuous activity but ambulatory and able to carry out light work; Performance status 2 = ambulatory and capable of all self-care but unable to carry out any work activities, up and about more than 50% of waking hour.

^d^ There were fewer than 10 patients in the “Never Married” group and no events had occurred, so the hazard ratio and 95% CI could not be estimated.

**Supplementary Table 2.** Overall survival by marital status stratified by clinicopathological and FFQ features.^a^

| **Subgroup** | **Count (%)** | **Married – HR** | **D/S/W – HR** | **D/S/W – *P-*value** | **Never-Married – HR** | **Never-Married – *P-*value** | ***P*_interaction_^b^** |
| --- | --- | --- | --- | --- | --- | --- | --- |
| ***Age (years)*** |  |  |  |  |  |  | 0.40 |
| ≤60 | 552(51.0) | ref | 1.75(1.18-2.60) | 0.005 | 1.27(0.73-2.20) | 0.39 |  |
| >60 | 530(49.0) | ref | 1.24(0.89-1.72) | 0.20 | 1.07(0.50-2.30) | 0.86 |  |
| ***Sex*** |  |  |  |  |  |  | 0.19 |
| Male | 605(55.9) | ref | 1.33(0.94-1.89) | 0.11 | 1.65(0.98-2.78) | 0.06 |  |
| Female | 477(44.1) | ref | 1.47(1.00-2.14) | 0.048 | 0.66(0.27-1.63) | 0.37 |  |
| ***Treatment arm*** |  |  |  |  |  |  | 0.97 |
| 5-FU/LV | 549(50.7) | ref | 1.40(1.00-1.98) | 0.05 | 1.15(0.60-2.22) | 0.67 |  |
| IFL | 533(49.3) | ref | 1.40(0.96-2.04) | 0.08 | 1.28(0.70-2.35) | 0.43 |  |
| ***T-stage*** |  |  |  |  |  |  | 0.99 |
| T1-2 ^d^ | 139(13.2) | ref | 1.35(0.51-3.59) | 0.54 | 0.00(0.00-23E246) | 0.97 |  |
| T3-4 | 917(86.8) | ref | 1.40(1.07-1.84) | 0.01 | 1.37(0.88-2.16) | 0.17 |  |
| ***Number of positive lymph nodes*** |  |  |  |  |  |  | 0.95 |
| 1-3 | 675(63.6) | ref | 1.37(0.95-1.98) | 0.10 | 1.23(0.70-2.15) | 0.48 |  |
| 4+ | 386(36.4) | ref | 1.43(1.00-2.04) | 0.049 | 1.40(0.67-2.90) | 0.37 |  |
| ***Performance status ^c^*** |  |  |  |  |  |  | 0.74 |
| ECOG0 | 794(75.0) | ref | 1.46(1.08-1.98) | 0.02 | 1.44(0.85-2.44) | 0.17 |  |
| ECOG1,2 | 264(25.0) | ref | 1.32(0.82-2.10) | 0.25 | 0.99(0.42-2.32) | 0.99 |  |
| ***Clinical bowel obstruction or perforation*** |  |  |  |  |  |  | 0.75 |
| No | 842(77.8) | ref | 1.44(1.08-1.93) | 0.01 | 1.13(0.66-1.93) | 0.66 |  |
| Yes | 240(22.2) | ref | 1.25(0.71-2.19) | 0.43 | 1.48(0.66-3.31) | 0.34 |  |
| ***Tumor location*** |  |  |  |  |  |  | 0.79 |
| Distal | 454(43.0) | ref | 1.32(0.84-2.05) | 0.23 | 1.07(0.51-2.23) | 0.86 |  |
| Proximal | 603(57.0) | ref | 1.43(1.04-1.97) | 0.03 | 1.45(0.82-2.56) | 0.20 |  |
| ***BMI*** |  |  |  |  |  |  | 0.35 |
| Min-24.9 | 346(33.1) | ref | 1.33(0.85-2.06) | 0.21 | 1.58(0.81-3.09) | 0.18 |  |
| 25-29.9 | 379(36.3) | ref | 1.78(1.12-2.80) | 0.01 | 1.23(0.49-3.09) | 0.65 |  |
| 30-max | 319(30.6) | ref | 1.11(0.71-1.73) | 0.64 | 0.65(0.26-1.63) | 0.36 |  |
| ***Physical activity*** |  |  |  |  |  |  | 0.28 |
| 0-2.9 met-h | 407(39.2) | ref | 1.20(0.82-1.76) | 0.35 | 0.78(0.37-1.61) | 0.50 |  |
| 3 met-h+ | 630(60.8) | ref | 1.51(1.06-2.16) | 0.02 | 1.56(0.85-2.85) | 0.15 |  |
| ***Western dietary pattern*** |  |  |  |  |  |  | 0.54 |
| <Median | 522(50.0) | ref | 1.39(0.95-2.03) | 0.09 | 0.88(0.42-1.80) | 0.72 |  |
| ≥Median | 522(50.0) | ref | 1.40(0.98-2.01) | 0.07 | 1.49(0.81-2.73) | 0.20 |  |
| ***Prudent dietary pattern*** |  |  |  |  |  |  | 0.23 |
| <Median | 522(50.0) | ref | 1.50(1.07-2.11) | 0.02 | 1.59(0.90-2.80) | 0.11 |  |
| ≥Median | 522(50.0) | ref | 1.22(0.81-1.84) | 0.33 | 0.70(0.30-1.60) | 0.39 |  |
| ***Household income*** |  |  |  |  |  |  | 0.23 |
| <Median | 413(49.9) | ref | 1.73(1.15-2.59) | 0.01 | 1.59(0.83-3.08) | 0.17 |  |
| ≥Median | 414(50.1) | ref | 1.08(0.70-1.66) | 0.73 | 0.97(0.41-2.25) | 0.94 |  |
| ***Insurance Status*** |  |  |  |  |  |  | 0.94 |
| Self-Pay/Private | 699(64.6) | ref | 1.46(1.03-2.09) | 0.04 | 1.26(0.71-2.21) | 0.43 |  |
| Medicare/Medicaid/Military/Other | 383(35.4) | ref | 1.34(0.93-1.93) | 0.12 | 1.16(0.56-2.40) | 0.69 |  |

Abbreviations: 5-FU = 5-fluorouracil; LV = leucovorin; IFL = irinotecan, 5-fluorouracil, leucovorin; FFQ = food frequency questionnaire; BMI = body mass index

^a^ Multivariable-adjusted model adjusted for age (continuous), sex (male, female), race (White, other), treatment arm, T-stage (T1-2, T3-4), number of positive nodes (1-3, 4+), performance status (ECOG 0, ECOG 1-2), tumor location (proximal, distal), clinical bowel obstruction or perforation (yes, no), consistent aspirin use (yes, no), insurance status (Self-Pay/Private, Medicare/Medicaid/Military/Other), valid FFQ1 (yes, no), household income, time-varying energy intake, BMI, physical activity, Western dietary pattern, prudent dietary pattern (all time-varying variables are continuous).

^b^ Interaction term built as a cross-product of marital status and the covariate of interest as binary variables.

^c^ Baseline performance status: Performance status 0 = fully active; Performance status 1 = restricted in physically strenuous activity but ambulatory and able to carry out light work; Performance status 2 = ambulatory and capable of all self-care but unable to carry out any work activities, up and about more than 50% of waking hour.

^d^ There were fewer than 10 patients in the “Never Married” group and no events had occurred, so the hazard ratio and 95% CI could not be estimated.

**Supplementary Table 3.** Disease-free survival by living arrangement stratified by clinicopathological and FFQ features.^a^

| **Subgroup** | **Count (%)** | **With spouse**  **/partner** | **Alone – HR** | **Alone – *P-*value** | **With other family – HR** | **With other family – *P-*value** | ***P*_interaction_^b^** |
| --- | --- | --- | --- | --- | --- | --- | --- |
| ***Age (years)*** |  |  |  |  |  |  | 0.51 |
| ≤60 | 545(51.0) | ref | 1.33(0.89-1.98) | 0.17 | 1.26(0.78-2.02) | 0.35 |  |
| >60 | 524(49.0) | ref | 1.21(0.87-1.69) | 0.26 | 1.84(1.07-3.17) | 0.03 |  |
| ***Sex*** |  |  |  |  |  |  | 0.81 |
| Male | 595(55.7) | ref | 1.17(0.80-1.69) | 0.42 | 1.32(0.74-2.36) | 0.34 |  |
| Female | 474(44.3) | ref | 1.32(0.92-1.91) | 0.13 | 1.60(1.00-2.54) | 0.05 |  |
| ***Treatment arm*** |  |  |  |  |  |  | 0.46 |
| 5-FU/LV | 542(50.7) | ref | 1.44(1.02-2.03) | 0.04 | 1.44(0.89-2.34) | 0.14 |  |
| IFL | 527(49.3) | ref | 1.05(0.721.54) | 0.81 | 1.51(0.89-2.55) | 0.13 |  |
| ***T-stage*** |  |  |  |  |  |  | 0.58 |
| T1-2 | 137(13.1) | ref | 0.59(0.14-2.49) | 0.48 | 1.30(0.30-5.53) | 0.73 |  |
| T3-4 | 907(86.9) | ref | 1.28(0.98-1.66) | 0.07 | 1.52(1.04-2.21) | 0.03 |  |
| ***Number of positive lymph nodes*** |  |  |  |  |  |  | 0.7 |
| 1-3 | 669(63.8) | ref | 1.12(0.78-1.61) | 0.54 | 1.45(0.91-2.32) | 0.12 |  |
| 4+ | 380(36.2) | ref | 1.39(0.97-2.01) | 0.08 | 1.54(0.88-2.69) | 0.13 |  |
| ***Performance status ^c^*** |  |  |  |  |  |  | 0.76 |
| ECOG0 | 787(75.2) | ref | 1.32(0.97-1.79) | 0.07 | 1.48(0.94-2.34) | 0.09 |  |
| ECOG1,2 | 259(24.8) | ref | 1.09(0.67-1.77) | 0.72 | 1.60(0.89-2.87) | 0.11 |  |
| ***Clinical bowel obstruction or perforation*** |  |  |  |  |  |  | 0.65 |
| No | 831(77.7) | ref | 1.17(0.87-1.57) | 0.31 | 1.49(0.98-2.25) | 0.06 |  |
| Yes | 238(22.3) | ref | 1.53(0.91-2.57) | 0.11 | 1.43(0.71-2.86) | 0.32 |  |
| ***Tumor location*** |  |  |  |  |  |  | 0.28 |
| Distal | 450(43.1) | ref | 0.90(0.55-1.47) | 0.67 | 1.56(0.91-2.69) | 0.11 |  |
| Proximal | 595(56.9) | ref | 1.41(1.03-1.92) | 0.03 | 1.42(0.87-2.31) | 0.16 |  |
| ***BMI*** |  |  |  |  |  |  | 0.17 |
| Min-24.9 | 341(33.0) | ref | 1.25(0.83-1.89) | 0.29 | 1.40(0.72-2.70) | 0.32 |  |
| 25-29.9 | 376(36.4) | ref | 1.79(1.13-2.83) | 0.01 | 1.34(0.64-2.78) | 0.44 |  |
| 30-max | 315(30.5) | ref | 0.76(0.45-1.27) | 0.29 | 1.52(0.87-2.66) | 0.14 |  |
| ***Physical activity*** |  |  |  |  |  |  | 0.64 |
| 0-2.9 met-h | 403(39.3) | ref | 1.15(0.78-1.69) | 0.49 | 1.21(0.71-2.08) | 0.48 |  |
| 3 met-h+ | 622(60.7) | ref | 1.25(0.87-1.81) | 0.23 | 1.72(1.03-2.86) | 0.04 |  |
| ***Western dietary pattern*** |  |  |  |  |  |  | 0.72 |
| <Median | 518(50.2) | ref | 1.25(0.86-1.80) | 0.24 | 1.61(1.00-2.62) | 0.05 |  |
| ≥Median | 514(49.8) | ref | 1.14(0.78-1.67) | 0.49 | 1.20(0.68-2.13) | 0.53 |  |
| ***Prudent dietary pattern*** |  |  |  |  |  |  | 0.86 |
| <Median | 514(49.8) | ref | 1.26(0.89-1.78) | 0.19 | 1.42(0.87-2.31) | 0.16 |  |
| ≥Median | 518(50.2) | ref | 1.10(0.73-1.64) | 0.66 | 1.45(0.82-2.55) | 0.2 |  |
| ***Household income*** |  |  |  |  |  |  | 0.29 |
| <Median | 407(49.7) | ref | 1.56(1.04-2.34) | 0.03 | 1.62(0.97-2.72) | 0.07 |  |
| ≥Median | 412(50.3) | ref | 0.98(0.63-1.51) | 0.91 | 1.62(0.90-2.91) | 0.11 |  |
| ***Insurance Status*** |  |  |  |  |  |  | 0.33 |
| Self-Pay/Private | 692(64.7) | ref | 1.16(0.82-1.65) | 0.4 | 1.21(0.75-1.94) | 0.44 |  |
| Medicare/Medicaid/Military/Other | 377(35.3) | ref | 1.36(0.93-2.00) | 0.12 | 2.04(1.18-3.53) | 0.01 |  |

Abbreviations: 5-FU = 5-fluorouracil; LV = leucovorin; IFL = irinotecan, 5-fluorouracil, leucovorin; FFQ = food frequency questionnaire; BMI = body mass index

^a^ Multivariable-adjusted model adjusted for age (continuous), sex (male, female), race (White, other), treatment arm, T-stage (T1-2, T3-4), number of positive nodes (1-3, 4+), performance status (ECOG 0, ECOG 1-2), tumor location (proximal, distal), clinical bowel obstruction or perforation (yes, no), consistent aspirin use (yes, no), insurance status (self-pay/private, Medicare/Medicaid/Military/Other), valid FFQ1 (yes, no), household income, time-varying energy intake, BMI, physical activity, Western dietary pattern, prudent dietary pattern (all time-varying variables are continuous).

^b^ Interaction term built as a cross-product of living arrangement and the covariate of interest as binary variables.

^c^ Baseline performance status: Performance status 0 = fully active; Performance status 1 = restricted in physically strenuous activity but ambulatory and able to carry out light work; Performance status 2 = ambulatory and capable of all self-care but unable to carry out any work activities, up and about more than 50% of waking hour.

**Supplementary Table 4.** Overall survival by living arrangement stratified by clinicopathological and FFQ features.^a^

| **Subgroup** | **Count (%)** | **With spouse**  **/partner** | **Alone – HR** | **Alone – *P-*value** | **With other family – HR** | **With other family – *P-*value** | ***P*_interaction_^b^** |
| --- | --- | --- | --- | --- | --- | --- | --- |
| ***Age (years)*** |  |  |  |  |  |  | 0.17 |
| ≤60 | 545(51.0) | ref | 1.50(0.96-2.33) | 0.07 | 1.09(0.61-1.96) | 0.76 |  |
| >60 | 524(49.0) | ref | 1.23(0.85-1.78) | 0.27 | 2.12(1.22-3.69) | 0.01 |  |
| ***Sex*** |  |  |  |  |  |  | 0.92 |
| Male | 595(55.7) | ref | 1.32(0.90-1.95) | 0.16 | 1.37(0.73-2.58) | 0.33 |  |
| Female | 474(44.3) | ref | 1.29(0.85-1.96) | 0.23 | 1.60(0.94-2.72) | 0.08 |  |
| ***Treatment arm*** |  |  |  |  |  |  | 0.86 |
| 5-FU/LV | 542(50.7) | ref | 1.39(0.95-2.05) | 0.09 | 1.47(0.86-2.50) | 0.16 |  |
| IFL | 527(49.3) | ref | 1.21(0.80-1.83) | 0.38 | 1.54(0.84-2.82) | 0.16 |  |
| ***T-stage*** |  |  |  |  |  |  | 0.65 |
| T1-2 | 137(13.1) | ref | 0.76(0.18-3.23) | 0.71 | 0.87(0.12-6.56) | 0.89 |  |
| T3-4 | 907(86.9) | ref | 1.34(1.00-1.79) | 0.05 | 1.60(1.05-2.44) | 0.03 |  |
| ***Number of positive lymph nodes*** |  |  |  |  |  |  | 0.84 |
| 1-3 | 669(63.8) | ref | 1.22(0.82-1.83) | 0.32 | 1.43(0.83-2.44) | 0.20 |  |
| 4+ | 380(36.2) | ref | 1.40(0.94-2.09) | 0.10 | 1.69(0.92-3.11) | 0.09 |  |
| ***Performance status ^c^*** |  |  |  |  |  |  | 0.59 |
| ECOG0 | 787(75.2) | ref | 1.43(1.03-2.00) | 0.03 | 1.49(0.88-2.52) | 0.14 |  |
| ECOG1,2 | 259(24.8) | ref | 1.06(0.62-1.83) | 0.83 | 1.66(0.88-3.15) | 0.12 |  |
| ***Clinical bowel obstruction or perforation*** |  |  |  |  |  |  | 0.98 |
| No | 831(77.7) | ref | 1.32(0.96-1.82) | 0.09 | 1.51(0.95-2.41) | 0.08 |  |
| Yes | 238(22.3) | ref | 1.23(0.66-2.31) | 0.52 | 1.46(0.66-3.22) | 0.35 |  |
| ***Tumor location*** |  |  |  |  |  |  | 0.27 |
| Distal | 450(43.1) | ref | 0.88(0.50-1.56) | 0.66 | 1.46(0.75-2.82) | 0.26 |  |
| Proximal | 595(56.9) | ref | 1.51(1.08-2.11) | 0.02 | 1.56(0.93-2.63) | 0.09 |  |
| ***BMI*** |  |  |  |  |  |  | 0.17 |
| Min-24.9 | 341(33.0) | ref | 1.38(0.88-2.18) | 0.16 | 1.88(0.93-3.80) | 0.08 |  |
| 25-29.9 | 376(36.4) | ref | 1.84(1.11-3.04) | 0.02 | 1.17(0.51-2.73) | 0.71 |  |
| 30-max | 315(30.5) | ref | 0.75(0.42-1.33) | 0.32 | 1.30(0.68-2.50) | 0.43 |  |
| ***Physical activity*** |  |  |  |  |  |  | 0.32 |
| 0-2.9 met-h | 403(39.3) | ref | 1.02(0.66-1.58) | 0.92 | 1.15(0.64-2.10) | 0.64 |  |
| 3 met-h+ | 622(60.7) | ref | 1.46(0.98-2.16) | 0.06 | 1.82(1.01-3.27) | 0.045 |  |
| ***Western dietary pattern*** |  |  |  |  |  |  | 0.55 |
| <Median | 518(50.2) | ref | 1.23(0.81-1.88) | 0.34 | 1.79(1.02-3.13) | 0.04 |  |
| ≥Median | 514(49.8) | ref | 1.34(0.90-1.98) | 0.15 | 1.17(0.63-2.18) | 0.63 |  |
| ***Prudent dietary pattern*** |  |  |  |  |  |  | 0.70 |
| <Median | 514(49.8) | ref | 1.40(0.96-2.03) | 0.08 | 1.48(0.85-2.57) | 0.16 |  |
| ≥Median | 518(50.2) | ref | 1.09(0.69-1.72) | 0.71 | 1.41(0.75-2.67) | 0.29 |  |
| ***Household income*** |  |  |  |  |  |  | 0.33 |
| <Median | 407(49.7) | ref | 1.66(1.07-2.58) | 0.02 | 1.75(1.00-3.04) | 0.05 |  |
| ≥Median | 412(50.3) | ref | 1.01(0.61-1.65) | 0.98 | 1.62(0.80-3.27) | 0.18 |  |
| ***Insurance Status*** |  |  |  |  |  |  | 0.14 |
| Self-Pay/Private | 692(64.7) | ref | 1.22(0.83-1.79) | 0.32 | 1.08(0.61-1.89) | 0.80 |  |
| Medicare/Medicaid/Military/Other | 377(35.3) | ref | 1.44(0.95-2.18) | 0.09 | 2.37(1.33-4.23) | 0.003 |  |

Abbreviations: 5-FU = 5-fluorouracil; LV = leucovorin; IFL = irinotecan, 5-fluorouracil, leucovorin; FFQ = food frequency questionnaire; BMI = body mass index

^a^ Multivariable-adjusted model adjusted for age (continuous), sex (male, female), race (White, other), treatment arm, T-stage (T1-2, T3-4), number of positive nodes (1-3, 4+), performance status (ECOG 0, ECOG 1-2), tumor location (proximal, distal), clinical bowel obstruction or perforation (yes, no), consistent aspirin use (yes, no), insurance status (self-pay/private, Medicare/Medicaid/Military/Other), valid FFQ1 (yes, no), household income, time-varying energy intake, BMI, physical activity, Western dietary pattern, prudent dietary pattern (all time-varying variables are continuous).

^b^ Interaction term built as a cross-product of living arrangement and the covariate of interest as binary variables.

^c^ Baseline performance status: Performance status 0 = fully active; Performance status 1 = restricted in physically strenuous activity but ambulatory and able to carry out light work; Performance status 2 = ambulatory and capable of all self-care but unable to carry out any work activities, up and about more than 50% of waking hour.
